# Effects of different doses of N-acetylcysteine on lung function in patients with chronic obstructive pulmonary disease: a meta-analysis

**DOI:** 10.1186/s12890-026-04523-5

**Published:** 2026-07-30

**Authors:** Yifan Wang, Xiaomin Chen, Jie Huang

**Affiliations:** 1 The Second School of Clinical Medicine, Zhejiang Chinese Medical University, Hang Zhou, Zhejiang Province People’s Republic Of China; 2 Department of Geriatrics, The Second Affiliated Hospital of Zhejiang Chinese Medical University (Xinhua Hospital of Zhejiang Province), Hangzhou, Zhejiang People’s Republic Of China

**Keywords:** COPD, Antioxidat, FEV_1_, FVC, FEV_1_/FVC

## Abstract

**Purpose:**

This meta-analysis seeks to examine the impact of different dosages of N-acetylcysteine (NAC) on pulmonary function in individuals with Chronic Obstructive Pulmonary Disease (COPD) and to clarify the dose-response relationship.

**Patients and methods:**

Systematic searches were performed in PubMed, the Wanfang Database, Cochrane Library, and China National Knowledge Infrastructure (CNKI) for pertinent randomized controlled trials (RCTs) published from 2000 to 2024. Research examining various dosages of N-acetylcysteine (NAC) for the management of chronic obstructive pulmonary disease (COPD) was incorporated. The methodological quality of the selected literature was evaluated utilizing the Cochrane Risk of Bias tool. This Meta-analysis was performed using RevMan software(version 5.4). The primary outcomes were lung function parameters, including forced expiratory volume in 1 s (FEV_1_), forced vital capacity (FVC), and the FEV_1_/FVC ratio.

**Results:**

High-dose NAC(≥ 1200 mg/d)demonstrated significant improvements in FEV_1_,FVC, and the FEV_1_/FVC ratio, whereas low-dose NAC(<1200 mg/d)showed nonsignificant effects. Substantial heterogeneity was observed for some outcomes. However, the efficacy of the high-dose regimen remained robust following sensitivity analysis.

**Conclusion:**

The findings of this study provide novel evidence supporting the differential effects of various NAC doses on improving lung function parameters in COPD patients. They suggest that high-dose NAC(≥ 1200 mg/d)may represent a preferred dosing strategy for enhancing lung function in COPD.

**PROSPERO registration number:**

CRD420251154781.

**Supplementary Information:**

The online version contains supplementary material available at https://doi.org/10.1186/s12890-026-04523-5.

## Background

Chronic obstructive pulmonary disease (COPD) is a widespread respiratory condition marked by persistent and progressively worsening airflow limitation. It currently stands as the third most common cause of death worldwide [1]. Common symptoms include a chronic cough, phlegm production, and increasing shortness of breath.

A major driver behind COPD’s progression is oxidative stress, largely triggered by exposure to cigarette smoke or other environmental pollutants [2]. In this context, N-acetylcysteine (NAC) has gained attention as a versatile antioxidant with mucolytic effects. It works both by directly neutralizing reactive oxygen species and by serving as a precursor to glutathione (GSH), a key intracellular antioxidant [3]. Through these mechanisms—boosting glutathione levels, clearing free radicals, and dampening inflammatory responses—NAC helps protect the airway lining from oxidative damage. These actions have been well-documented in several reviews [4, 5].

Clinically, NAC has often been studied for its potential to reduce acute exacerbations of COPD. Some trials have also explored its role in easing respiratory symptoms across different stages of disease severity [6]. That said, there remains a noticeable gap in systematic reviews and meta-analyses evaluating how different dosages of NAC may influence lung function improvement in COPD patients [7, 8].

Although NAC is widely used as adjunctive therapy for COPD, no clear consensus exists on its optimal dosage. The Chinese Guidelines for the Prevention and Treatment of Chronic Obstructive Pulmonary Disease (2023 Edition) recommends 600 mg/day primarily for preventing exacerbations. The GOLD 2023 guidelines do not specify a dosage, merely suggesting NAC as a supplementary antioxidant therapy(2).

The considerable variation in NAC dosing (600–1800 mg/day) across trials has contributed to inconsistent efficacy outcomes [9]. A clear understanding of how dosage influences the improvement of lung function—a critical endpoint for COPD patients—is still missing from the literature.

To address this gap, we conducted a dose-stratified meta-analysis. Our primary objective was to determine the specific effects of low versus high-dose NAC regimens on spirometric parameters (FEV_1_, FVC, FEV_1_/FVC). We sought to establish a robust evidence base to inform more precise and effective dosing recommendations for clinical practice.

## Materials and methods

This study established inclusion and exclusion criteria based on the PICOS principles as follows:

Inclusion Criteria: The study type selected is randomized controlled trials (RCTs) evaluating N-acetylcysteine (NAC) for the treatment of chronic obstructive pulmonary disease (COPD). All study subjects are patients with confirmed chronic obstructive pulmonary disease (COPD), regardless of gender or disease status (stable or acute exacerbation).

Interventions:

- Experimental groups administered varying doses of N-acetylcysteine (NAC). Based on previous meta-analyses and clinical trial evidence, NAC doses were categorized as high-dose (≥ 1200 mg/day) and low-dose (< 1200 mg/day). The threshold of 1200 mg/day was selected based on the following rationale:

First, a seminal meta-analysis by Cazzola et al. (2015) demonstrated that high-dose NAC (≥ 1200 mg/day) was effective in preventing exacerbations in COPD patients with documented airway obstruction, whereas low-dose NAC (≤ 600 mg/day) was sufficient only for chronic bronchitis without airway obstruction [10]. Second, the PANTHEON study, a large-scale randomized controlled trial, specifically used 1200 mg/day (600 mg twice daily) to evaluate NAC’s efficacy in preventing COPD exacerbations [11] .Third, multiple systematic reviews have consistently adopted 1200 mg/day as the threshold for “high-dose” NAC in COPD, as doses below this level (typically 600 mg/day) have shown inconsistent or non-significant effects on clinical outcomes [12, 13]. Fourth, a network meta-analysis by Cazzola et al. (2017) confirmed that NAC at 1200 mg/day significantly protected against COPD exacerbations, while 600 mg/day did not [14] .Therefore, the 1200 mg/day cutoff represents an evidence-based threshold supported by both clinical trial data.

Study results:

- Provided extractable continuous variables (mean, SD) or data estimable from graphs;

Outcome measures:

- Reported at least one pulmonary function parameter, including FEV_1_ (L), FVC (L), or FEV_1_/FVC (%);

Exclusion criteria:

- if they were not RCTs; if the administered NAC dosage was unclear or could not be compared between groups; if NAC was co-administered with other antioxidants, which would make it impossible to isolate its independent effect; if they failed to report relevant pulmonary function outcomes or provided data that could not be extracted; if they were duplicate publications (in which case, only the most complete report was included); or if they were not published in English or Chinese, or if the full text could not be obtained.

No data conversion or imputation was necessary for the included studies, as all required data were reported directly.

### Ethics statement

This study is a meta-analysis based on previously published studies; therefore, ethical approval and patient consent were not required.

### Research methods

Computer-based retrieval of relevant literature on N-acetylcysteine (NAC) treatment for chronic obstructive pulmonary disease (COPD) from PubMed, the Wanfang Database, Cochrane Library, and China National Knowledge Infrastructure (CNKI). The search was restricted to databases up to December 2024,and the last search time is September 2025.The search language was set to English and Chinese. For foreign databases, the search terms were “COPD, Antioxidant, RCT” for Chinese databases, the search terms were “COPD, N-acetylcysteine”. In Pubmed, the full search strategies is “((((((((((((COPD) OR (Pulmonary Disease, Chronic Obstructive)) OR (Chronic Obstructive Pulmonary Diseases)) OR (Chronic Obstructive Lung Disease)) OR (Chronic Obstructive Pulmonary Disease)) OR (COAD)) OR (Chronic Obstructive Airway Disease)) OR (Airflow Obstruction, Chronic)) OR (Airflow Obstructions, Chronic)) OR (Chronic Airflow Obstructions)) OR (Chronic Airflow Obstruction)) AND ((((((((((((((((((Antioxidant) OR (Anti-Oxidant)) OR (Anti Oxidant)) OR (Anti-Oxidants)) OR (Anti Oxidants)) OR (Antioxidant)) OR (Antioxidant Effect)) OR (Antioxidant Effects)) OR (Anti-Oxidant Effect)) OR (Anti Oxidant Effect)) OR (Anti-Oxidant Effects)) OR (Anti Oxidant Effects)) OR (Antioxidant Activity)) OR (Activity, Antioxidant)) OR (Endogenous Antioxidants)) OR (Antioxidants, Endogenous)) OR (Endogenous Antioxidant)) OR (Antioxidant, Endogenous))) AND ((RCT) OR (Meta-analysis))”.

The literature search combined subject headings with free-text terms, while also performing synonym expansion. To prevent omissions, further searches were conducted of relevant references.

Two researchers independently screened the literature according to inclusion and exclusion criteria. In cases of disagreement, a third researcher participated in the discussion to reach a decision. Basic study information, sample size, intervention measures, and outcome measures were extracted.

Two researchers independently assessed study quality using the Cochrane 5.1.0 Manual for Systematic Reviews [15]. Disagreements were resolved through discussion with a third researcher to determine overall study quality. Evaluation criteria included random sequence generation, allocation concealment, blinding, completeness of outcome data, selective reporting of results, and other sources of bias. Based on these criteria, included studies were rated as “high risk of bias,” “low risk of bias,” or “unknown risk of bias.” Literature quality was categorized into three grades: Grade A (studies fully meeting the criteria with minimal bias in results), Grade B (partially meeting criteria with moderate bias), and Grade C (failing to meet criteria with high bias).

### GRADE assessment

The quality of evidence for each outcome (FEV₁, FVC, and FEV₁/FVC) was assessed using the Grading of Recommendations Assessment, Development and Evaluation (GRADE) approach (Table 1). For each outcome, evidence from randomized controlled trials started as high quality and could be downgraded based on five domains: risk of bias, inconsistency, indirectness, imprecision, and publication bias. The GRADE assessment was performed independently by two reviewers, with disagreements resolved through consensus. A summary of findings table was generated for each outcome.

**Table 1:** GRADE summary of findings: n-acetylcysteine compared to placebo or usual care for patients with chronic obstructive pulmonary disease

Outcomes	№ of participants (studies)	Effect estimate (MD, 95% CI)	Risk of bias	Inconsistency	Indirectness	Imprecision	Publication bias	Overall certainty of evidence (GRADE)
FEV₁ (L)	2209 (11 RCTs)	MD 0.13 higher (0.07 to 0.20)	Serious¹	Serious²	Not serious	Serious³	Not detected⁴	⊕○○○ **VERY LOW**
FVC (L)	1887 (7 RCTs)	MD 0.17 higher (0.02 to 0.32)	Serious¹	Not serious⁵	Not serious	Not serious	Not detected⁴	⊕⊕⊕○ **MODERATE**
FEV₁/FVC (%)	1958 (8 RCTs)	MD 2.53 higher (0.54 to 4.52)	Serious¹	Not serious⁶	Not serious	Not serious	Not detected⁴	⊕⊕⊕○ **MODERATE**

*Abbreviations*: *CI* confidence interval, *FEV₁* forced expiratory volume in 1 s, *FVC* forced vital capacity, *MD* mean difference, *RCT* randomized controlled trial

GRADE Working Group grades of evidence: High certainty: We are very confident that the true effect lies close to that of the estimate of the effect

Moderate certainty: We are moderately confident in the effect estimate: The true effect is likely to be close to the estimate of the effect, but there is a possibility that it is substantially different

Low certainty: Our confidence in the effect estimate is limited: The true effect may be substantially different from the estimate of the effect

Very low certainty: We have very little confidence in the effect estimate: The true effect is likely to be substantially different from the estimate of effect

Explanations: ¹ Risk of bias: Among the included studies, quality distribution was as follows: FEV₁ (6 low-risk, 2 moderate-risk, 3 high-risk); FVC (3 low-risk, 2 moderate-risk, 2 high-risk); FEV₁/FVC (4 low-risk, 3 moderate-risk, 1 high-risk). The presence of moderate- and high-risk studies warranted downgrading by one level

² Inconsistency: Substantial heterogeneity was observed for FEV₁ (I² = 97%). Sensitivity analysis did not resolve the heterogeneity, with overall I² remaining at 96%

³ Imprecision: After sensitivity analysis, the 95% confidence interval for FEV₁ crossed the null value ([-0.01, 0.12]) and lost statistical significance (*P* = 0.07), warranting downgrading by one level

⁴ Publication bias: Funnel plots for FEV₁ and FVC were symmetric. Although the funnel plot for FEV₁/FVC showed potential asymmetry, formal testing was not performed due to the limited number of studies (< 10), and no downgrade was applied

⁵ Inconsistency: After sensitivity analysis, heterogeneity for FVC was substantially reduced (I² decreased from 91% to 9%)

⁶ Inconsistency: After sensitivity analysis, heterogeneity for FEV₁/FVC was substantially reduced (I² decreased from 90% to 21%)

### Statistical analysis

Statistical analysis was performed using RevMan 5.4 software. All outcome measures were continuous variables, with effect sizes represented by weighted mean differences (MD) or standardized mean differences (SMD) and their 95% confidence intervals (95% CI). Heterogeneity was assessed using Cochran’s Q test and the I² statistic: when I² < 50%, low heterogeneity was assumed, and a fixed-effect model was used; when I² ≥ 50%, substantial heterogeneity was indicated, and a random-effects model was employed for meta-analysis.

To further explore the relationship between dose and efficacy, a subgroup analysis stratified by dose was conducted (high dose ≥ 1200 mg/d, low dose < 1200 mg/d). Additionally, to validate the robustness of the results, a sensitivity analysis was performed by excluding studies with high risk of bias to observe changes in the analysis results. A p-value < 0.05 was considered statistically significant.

### Literature search results

Through initial database searches, 804 records were identified, comprising 48 English articles from PubMed, 263 Chinese articles from CNKI, 265 English articles from the Cochrane Library, and 226 Chinese/English articles from the Wanfang Database. After deduplication, 545 articles proceeded to the title and abstract screening stage. During preliminary screening, 493 articles were excluded for not meeting the inclusion criteria ( non-RCTs, irrelevant interventions, duplicate publications), leaving 52 articles for full-text assessment. After detailed full-text review, 40 studies were excluded due to lack of quantitative lung function data (FEV₁, FVC, or FEV₁/FVC). Ultimately, 12 eligible randomized controlled trials (RCTs) were included in the meta-analysis.

## Literature screening process (Fig. 1)

**Fig. 1:**
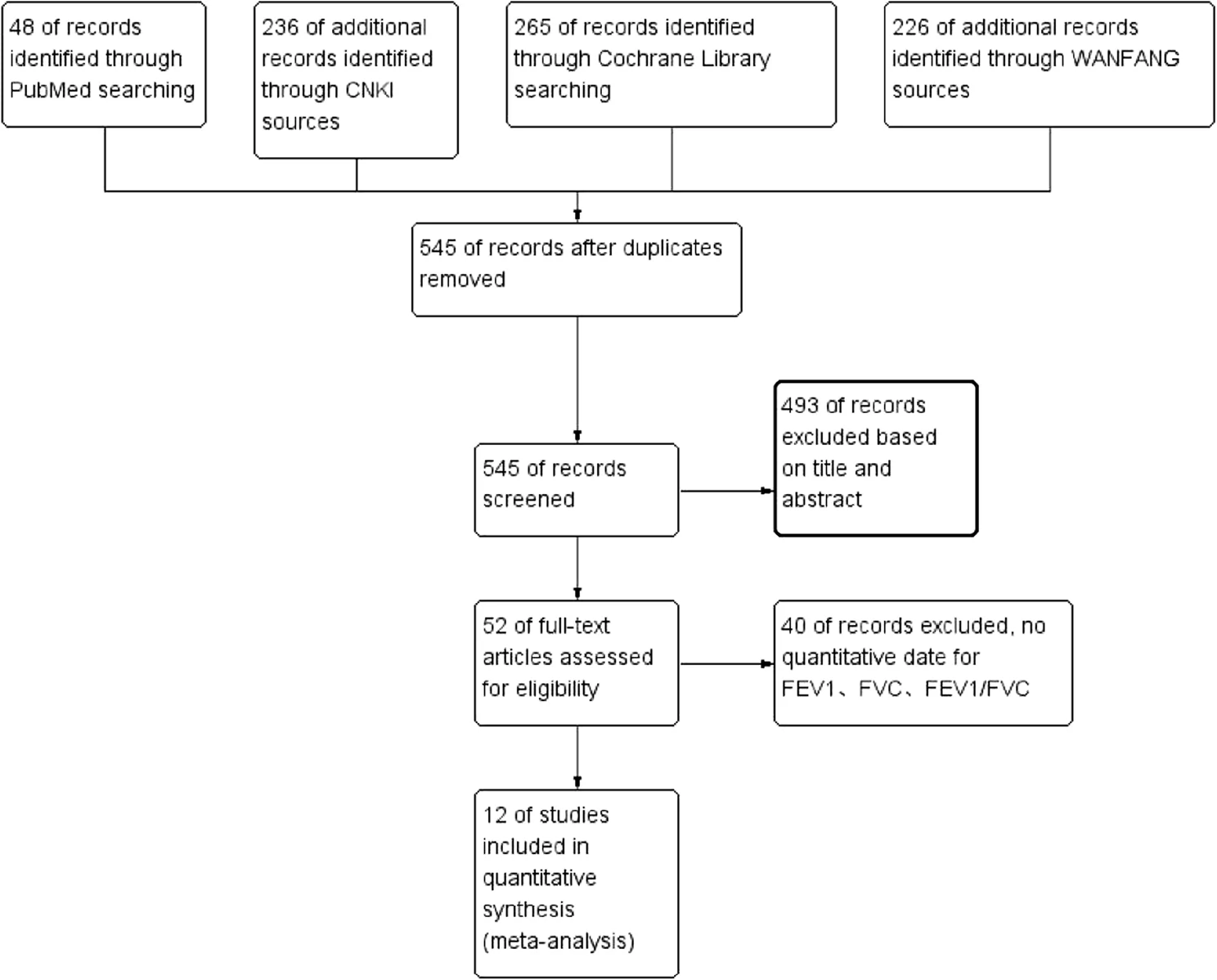
PRISMA flow diagram of study selection process

### Basic characteristics and methodological quality assessment of included studies

The basic characteristics of the included studies are presented in Table 2. The quality of the included studies was assessed according to the Cochrane 5.1.0 systematic review manual, with 8 studies rated as Grade A and 4 studies rated as Grade B. The included studies spanned the period from 2000 to 2024. There were no statistically significant differences (*P* > 0.05) between the experimental and control groups in baseline characteristics such as age, gender ratio, smoking history, and baseline pulmonary function indicators (, FEV_1_, FVC, FEV_1_/FVC), indicating comparability. The methodological quality assessment of the included studies is presented in Table 3.

**Table 2:** Basic characteristics table

Author (Year)	Country	Study Design	Sample Size (Intervention/Control)	NAC Dose (mg/day)	Duration	COPD Severity (GOLD)	Outcome Measures	Main Findings
Zhou, Y 2024	China	RCT	924 (464/460)	High: 1200	24month	GOLD1-2	FEV1,FVC, FEV1/FVC	No significant difference
Kolarov 2022	Serbia	RCT	116 (37/37/42)	Low: 600 High: 1200	6month	GOLD1-4	FEV1/FVC	No significant difference
Johnson 2016	USA	RCT	45(23/22)	High: 1800	8week	GOLD1-4	FEV1,FVC, FEV1/FVC	No significant difference
Ayfer Aytemur2015	Turkey	RCT	38(19/19)	Low: 600	12month	GOLD1-4	FEV1	No significant difference
Tse2013	China	RCT	120(58/62)	High: 1200	12month	GOLD1-4	FEV1,FVC	No significant difference
Schermer2009	Netherlands	RCT	192(96/96)	Low: 600	36month	GOLD1-4	FEV1	No significant difference
Liu, Y.2022	China	RCT	400(200/200)	High: 1200	12month	GOLD1-4	FEV1,FVC, FEV1/FVC	No significant difference
Wang, J.2018	China	RCT	120(40/40/40)	Low: 600 High: 1200	6month	GOLD1-4	FEV1,FVC	No significant difference
Ren, B.C.2016	China	RCT	158(79/79)	High: 1800	12month	GOLD1-4	FEV1,FVC, FEV1/FVC	No significant difference
Wei, G.L.2008	China	RCT	93(50/43)	Low: 600	6month	GOLD1-4	FEV1,FEV1/FVC	No significant difference
He, X.H. 2024	China	RCT	80(40/40)	Low: 600	12month	GOLD2-4	FEV1, FVC, FEV1/FVC	No significant difference
Miao, S.2018	China	RCT	100(51/49)	Low: 600	4week	GOLD1-4	FEV1/FVC	No significant difference

*Abbreviations*: *FEV₁* forced expiratory volume in 1 s (measured in liters), *FVC* forced vital capacity (measured in liters), *FEV₁/FVC* ratio of forced expiratory volume in 1 s to forced vital capacity (measured as a percentage)

**Table 3:** Quality evaluation form

Author (Year)	Random Sequence Generation	Allocation Concealment	Blinding of Participants	Blinding of Outcome Assessors	Incomplete Outcome Data	Selective Reporting	Other Bias	Overall Risk of Bias
Zhou, Y 2024	Low Risk	Low Risk	Low Risk	Low Risk	Low Risk	Low Risk	None	Moderate
Kolarov 2022	Low Risk	Low Risk	Low Risk	Low Risk	Low Risk	Low Risk	None	Moderate
Johnson 2016	Low Risk	Low Risk	Low Risk	Low Risk	Low Risk	Low Risk	None	Moderate
Ayfer Aytemur2015	Low Risk	Low Risk	Low Risk	Low Risk	Low Risk	Low Risk	None	Moderate
Tse2013	Low Risk	Low Risk	Low Risk	Unclear Risk	Low Risk	Low Risk	None	Moderate
Schermer2009	Low Risk	Low Risk	Low Risk	Unclear Risk	Low Risk	Low Risk	None	Moderate
Liu, Y.2022	Low Risk	Low Risk	Unclear Risk	Unclear Risk	Low Risk	Low Risk	None	Moderate
Wang, J.2018	Low Risk	Low Risk	Unclear Risk	Unclear Risk	Low Risk	Low Risk	None	Moderate
Ren, B.C.2016	Low Risk	Low Risk	Unclear Risk	Low Risk	Low Risk	Low Risk	None	Moderate
Wei, G.L.2008	Low Risk	Low Risk	Unclear Risk	Unclear Risk	Low Risk	Low Risk	None	Moderate
He, X.H. 2024	Low Risk	Low Risk	Low Risk	Unclear Risk	Low Risk	Low Risk	None	Moderate
Miao, S.2018	Low Risk	Low Risk	Unclear Risk	Unclear Risk	Low Risk	Low Risk	None	Moderate

*Abbreviations*: *SD* Standard Deviation, *MD* Mean Difference

### Assessment of reporting biases

Due to the limited number of studies in each meta-analysis (< 10), the statistical power to detect publication bias was low. Therefore, formal statistical testing was not performed, as recommended in the Cochrane Handbook.

## GRADE assessment

The certainty of evidence for each primary outcome was assessed using the GRADE approach (Table 1). For FEV₁, the overall certainty of evidence was rated as very low due to serious risk of bias, serious inconsistency, and serious imprecision after sensitivity analysis. For FVC and FEV₁/FVC, the overall certainty of evidence was rated as moderate, downgraded only for serious risk of bias, with inconsistency resolved after sensitivity analysis. No downgrade was applied for indirectness or publication bias.

## Results

### Effect of NAC on FEV_1_

The meta-analysis comparing the effects of different NAC doses on FEV_1_ (L)in COPD patients included 11 studies, involving 1,109 subjects in the intervention group and 1,100 in the control group. Meta-analysis results showed: In the high-dose group (≥ 1200 mg/d), NAC intervention significantly improved FEV_1_, with a pooled effect size of MD = 0.19, 95% CI [0.02, 0.37], *P* = 0.03, indicating a statistically significant difference. This subgroup exhibited high heterogeneity (I² = 96%). In the low-dose group (<1200 mg/day), NAC intervention did not significantly improve FEV₁, with a pooled effect size of MD = 0.05, 95% CI [–0.11, 0.20], *P* = 0.56, indicating no statistically significant difference. Heterogeneity was present (I² = 90%).

The overall pooled analysis demonstrated that NAC intervention at various doses generally improved FEV_1_ (MD = 0.13, 95% CI [0.07, 0.20], *P* < 0.0001), though with substantial heterogeneity (I² = 97%).

**Fig. 2:**
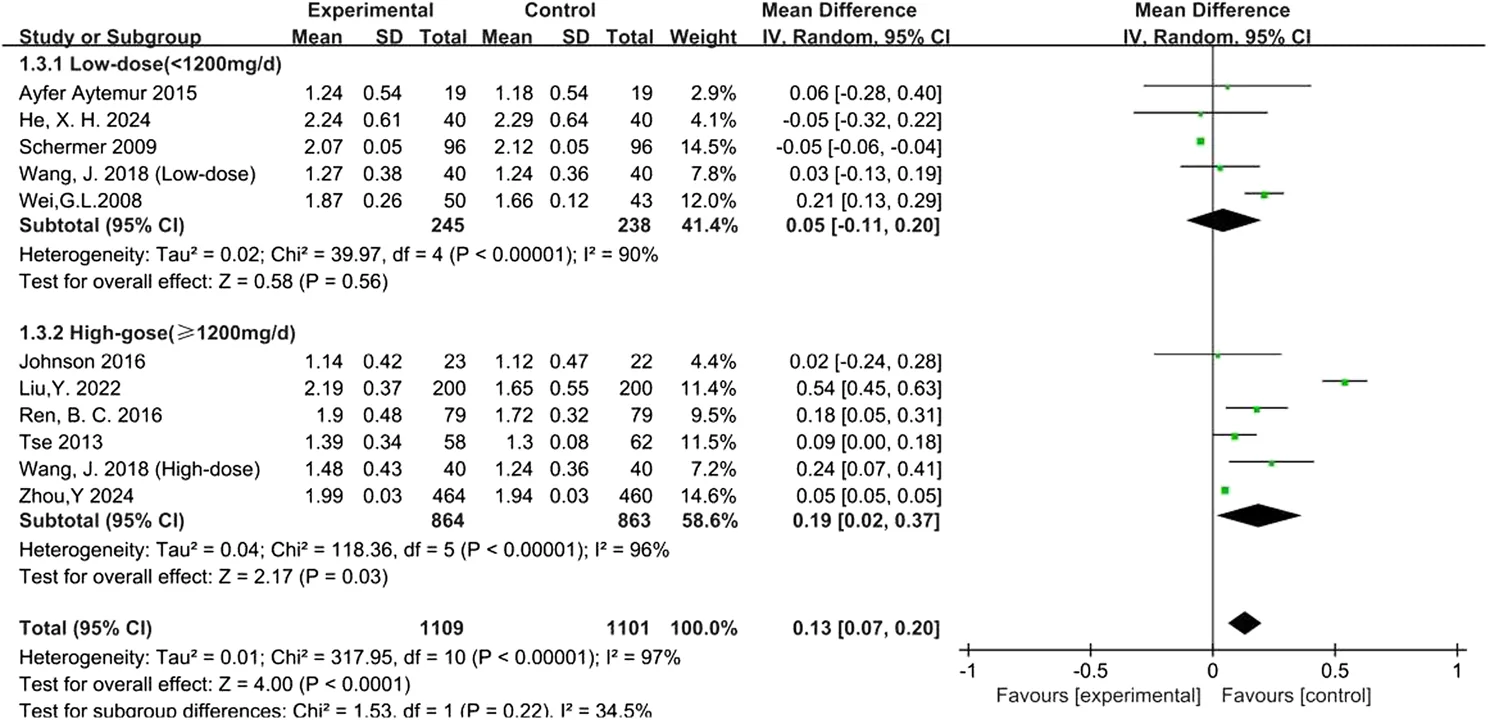
Forest plot of the effect of NAC on FEV_1_ in COPD patients

Although the high-dose group demonstrated a significant within-group effect, the test for subgroup differences indicated that the difference between the high-dose and low-dose groups was not statistically significant (Chi² = 1.53, *P* = 0.22;). (Fig. 2)

### Effect of NAC on FVC

A total of 7 studies were included (944 patients in the intervention group and 943 in the control group), comparing the effects of different NAC doses on FVC(L) in COPD patients: In the high-dose group (≥ 1200 mg/d), NAC significantly increased patients’ FVC levels, with a pooled effect size of MD = 0.22, 95% CI [0.05, 0.40], *P* = 0.01, indicating a statistically significant difference. This subgroup exhibited high heterogeneity (I²=93%). In the low-dose group (<1200 mg/d), NAC showed no statistically significant improvement in FVC (MD = 0.01, 95% CI [–0.17, 0.18], *P* = 0.95), with low heterogeneity (I²=0%).

Overall pooled results indicated that NAC intervention significantly increased FVC (MD = 0.17, 95% CI [0.02, 0.32], *P* = 0.02), but with high heterogeneity (I²=91%).

**Fig. 3:**
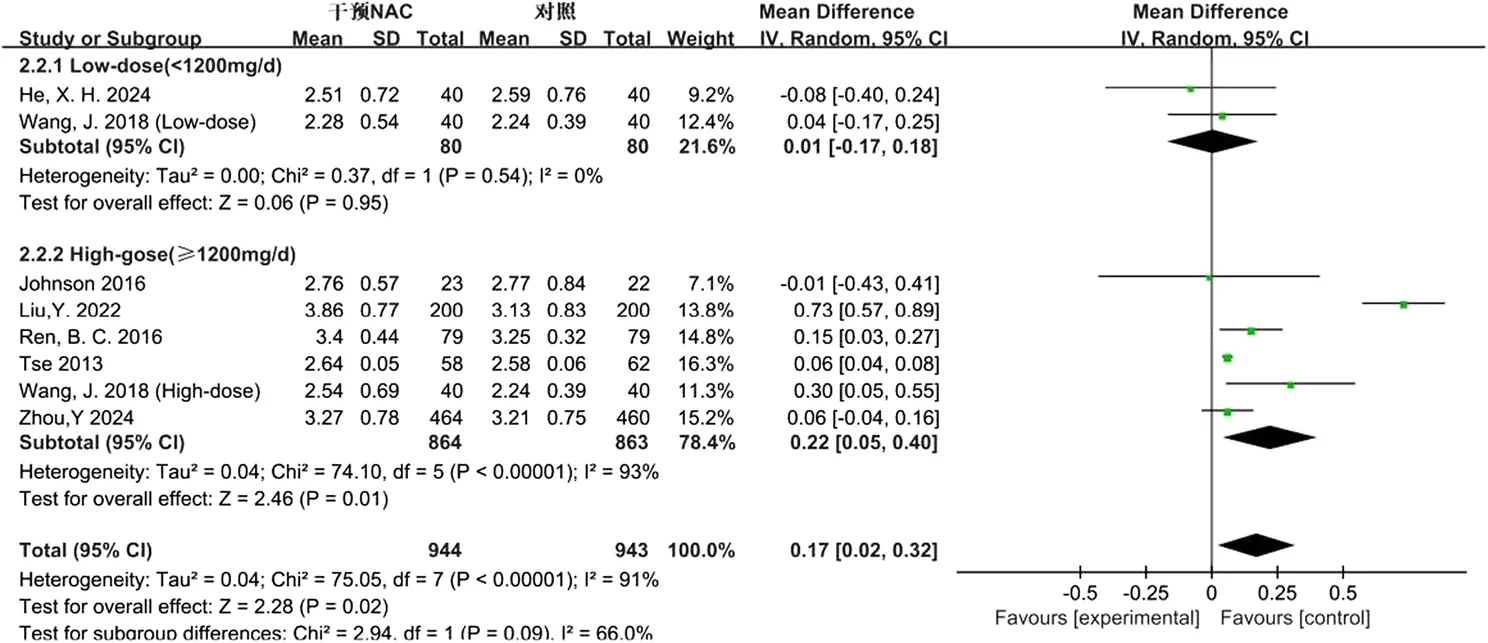
Forest plot of the effect of NAC on FVC in COPD patients

Subgroup comparisons indicated a significant difference between high- and low-dose NAC in improving FVC (Chi²=2.94, *P* = 0.09), though this did not meet statistical significance criteria. (Fig. 3)

### Effect of NAC on FEV_1_/FVC

A total of 8 studies (981 patients in the intervention group and 977 in the control group) were included to evaluate the effect of NAC on the FEV_1_/FVC(%) ratio in COPD patients. The results are as follows: In the low-dose group (<1200 mg/day), NAC demonstrated statistically significant improvement in FEV_1_/FVC with a pooled effect size of MD = 2.76, 95% CI [0.09, 5.43], *P* = 0.04, though heterogeneity was high (I² = 79%). In the high-dose group (≥ 1200 mg/d), although the pooled effect size was MD = 2.30, 95% CI [–0.89, 5.49], the difference did not reach statistical significance (*P* = 0.16), with similarly high heterogeneity (I² = 94%).

Overall pooled results indicated that NAC intervention significantly improved the FEV_1_/FVC ratio, with MD = 2.53, 95% CI [0.54, 4.52], *P* = 0.01, suggesting statistical significance; however, substantial heterogeneity persisted (I² = 90%).

**Fig. 4:**
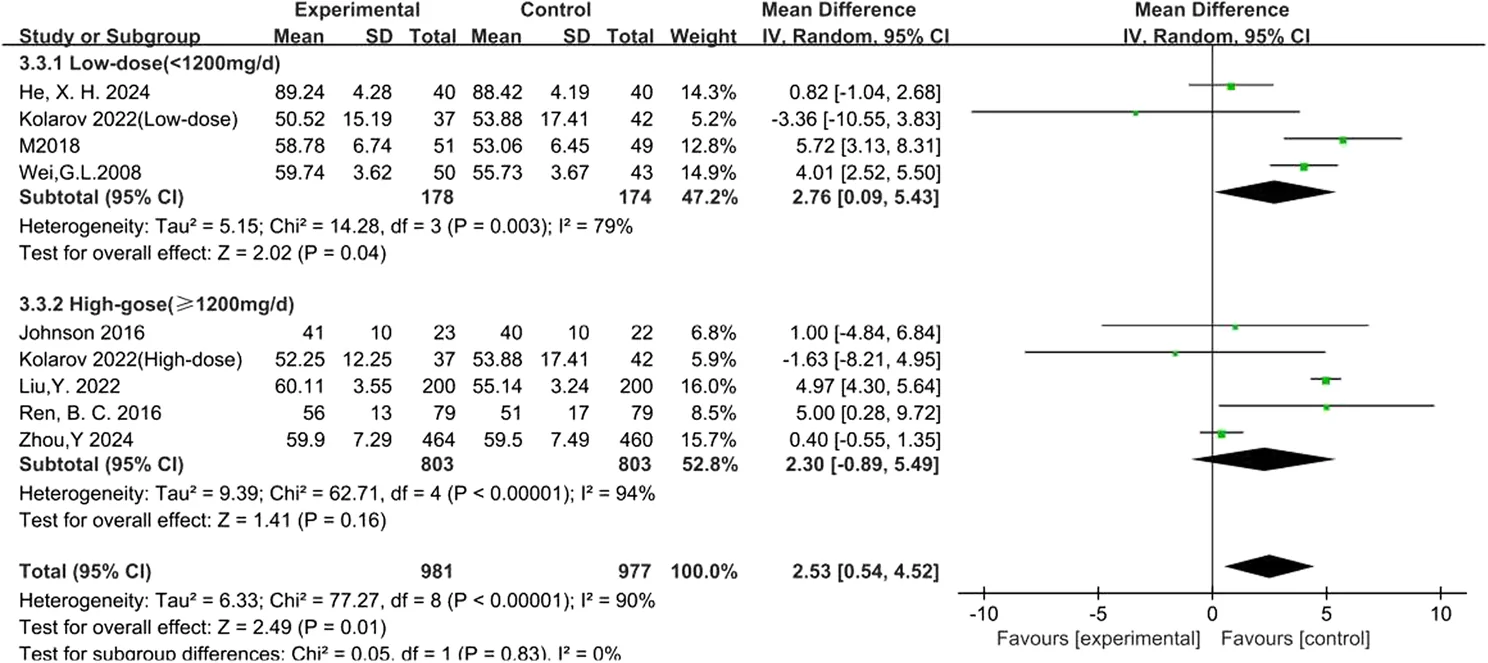
Forest plot of the effect of NAC on FEV_1_/FVC ratio in COPD patients

Subgroup comparisons revealed no statistically significant difference between high- and low-dose groups (Chi² = 0.05, *P* = 0.83). (Fig. 4)

## Sensitivity analysis

To explore the robustness of primary outcome analysis results and potential sources of heterogeneity, this study conducted sensitivity analyses for three pulmonary function indicators: FEV_1_, FVC, and FEV_1_/FVC. By excluding studies with a high risk of bias or significant contributions to heterogeneity, the pooled effect sizes were recalculated and dose-stratified comparisons performed. The results are as follows.

### Sensitivity analysis of FEV_1_

To explore sources of heterogeneity and validate the robustness of results, this study conducted a sensitivity analysis on FEV_1_, re-pooling effects after excluding studies with high heterogeneity.

In the high-dose group (≥ 1200 mg/d), NAC intervention still significantly improved FEV_1_ (MD = 0.10, 95% CI [0.03, 0.17], *P* = 0.004), with heterogeneity reduced to moderate levels (I² = 57%). In the low-dose group (< 1200 mg/d), the pooled effect size was MD = -0.05 (95%CI [-0.06, -0.04], *P* < 0.00001), indicating that NAC intervention did not show an improvement in FEV₁.

The overall pooled results showed that the improvement effect of NAC on FEV_1_ was no longer statistically significant (MD = 0.06, 95% CI [–0.01, 0.12], *P* = 0.07), suggesting that the original findings may have been influenced by studies with high heterogeneity.

**Fig. 5:**
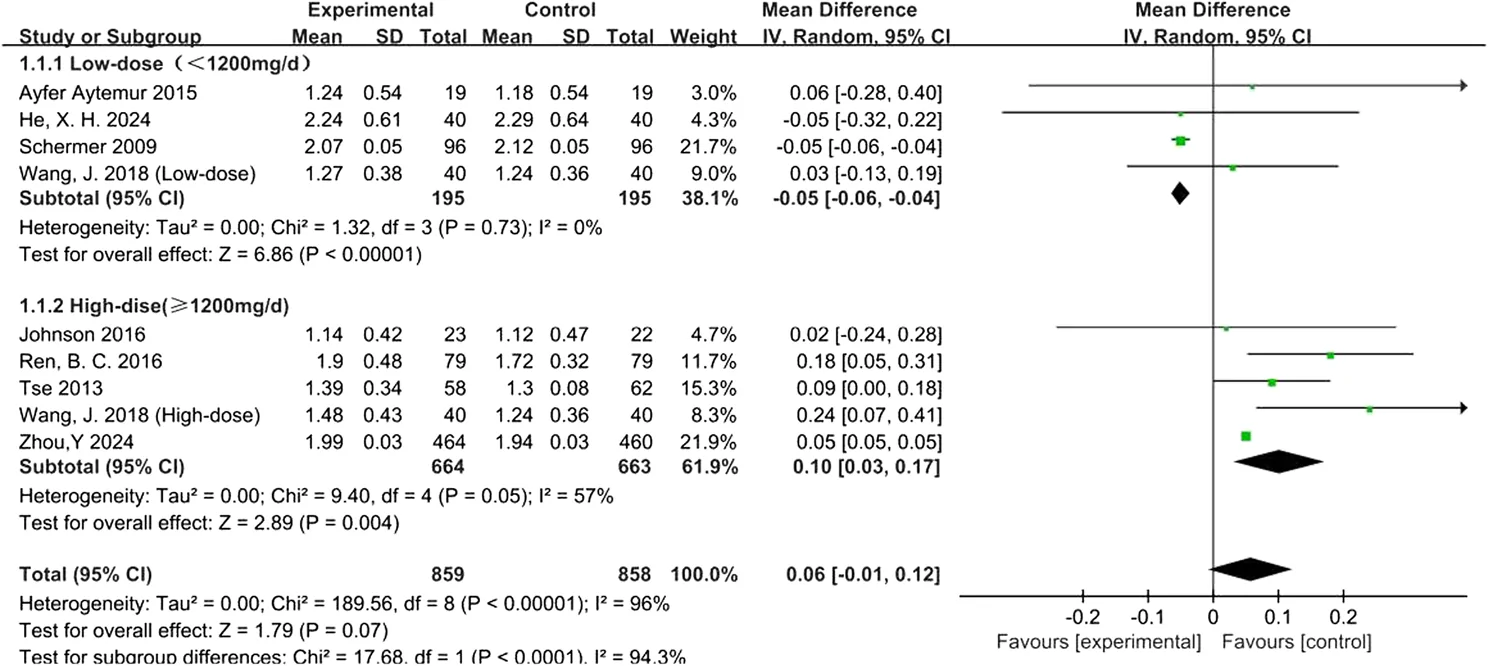
Sensitivity analysis of the effect of NAC on FEV_1_ in COPD patients (by dosage subgroup)

Subgroup differences were statistically significant (Chi² = 17.68, *P* < 0.0001), suggesting a potential true difference in FEV₁ effects across different doses. (Fig. 5)

### Sensitivity analysis of FVC

For the FVC indicator, the sensitivity analysis included 5 studies with a total of 744 patients in the intervention group and 743 in the control group. The results are as follows:

In the high-dose group (≥ 1200 mg/d), NAC significantly improved FVC with a pooled effect size of MD = 0.08, 95% CI [0.03, 0.14], *P* = 0.003, and markedly reduced heterogeneity (I² = 31%), indicating robust results. In the low-dose group (<1200 mg/d), the intervention effect was not statistically significant (MD = 0.01, 95% CI [–0.17, 0.18], *P* = 0.95), with 0% heterogeneity.

The overall pooled analysis demonstrated that NAC significantly improved FVC (MD = 0.07, 95% CI [0.03, 0.11], *P* = 0.0002) with low heterogeneity (I² = 9%), indicating robust results.

**Fig. 6:**
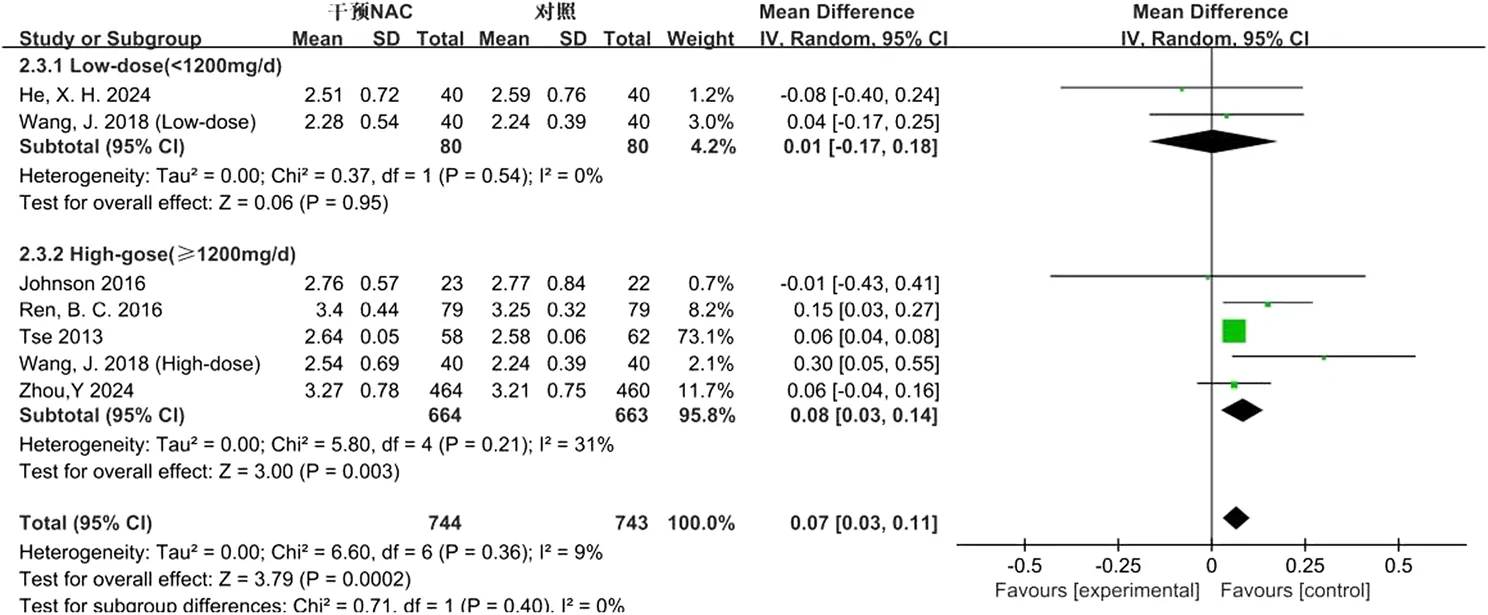
Sensitivity analysis of the effect of NAC on FVC in COPD patients (by dosage subgroup)

No significant differences were observed between subgroups (Chi² = 0.71, P = 0.40). (Fig. 6).

#### Sensitivity analysis of FEV_1_/FVC

To validate the robustness of FEV_1_/FVC analysis results and reduce heterogeneity, this study conducted a sensitivity analysis on this indicator, incorporating a total of 7 studies (440 cases in the intervention group, 435 cases in the control group).

In the low-dose group (<1200 mg/d), NAC intervention showed a statistically significant improvement in the FEV_1_/FVC ratio (MD = 4.52, 95% CI [2.99, 6.06], *P* < 0.00001) with low heterogeneity (I² = 21%). In the high-dose group (≥ 1200 mg/d), NAC also significantly increased FEV1/FVC (MD = 3.54, 95% CI [0.86, 6.22], *P* = 0.01) with moderate heterogeneity (I² = 46%).

Overall pooled results demonstrated that NAC significantly improved FEV_1_/FVC (MD = 4.52, 95% CI [3.46, 5.57], *P* < 0.00001) with low heterogeneity (I² = 21%), indicating robust and reliable findings.

**Fig. 7:**
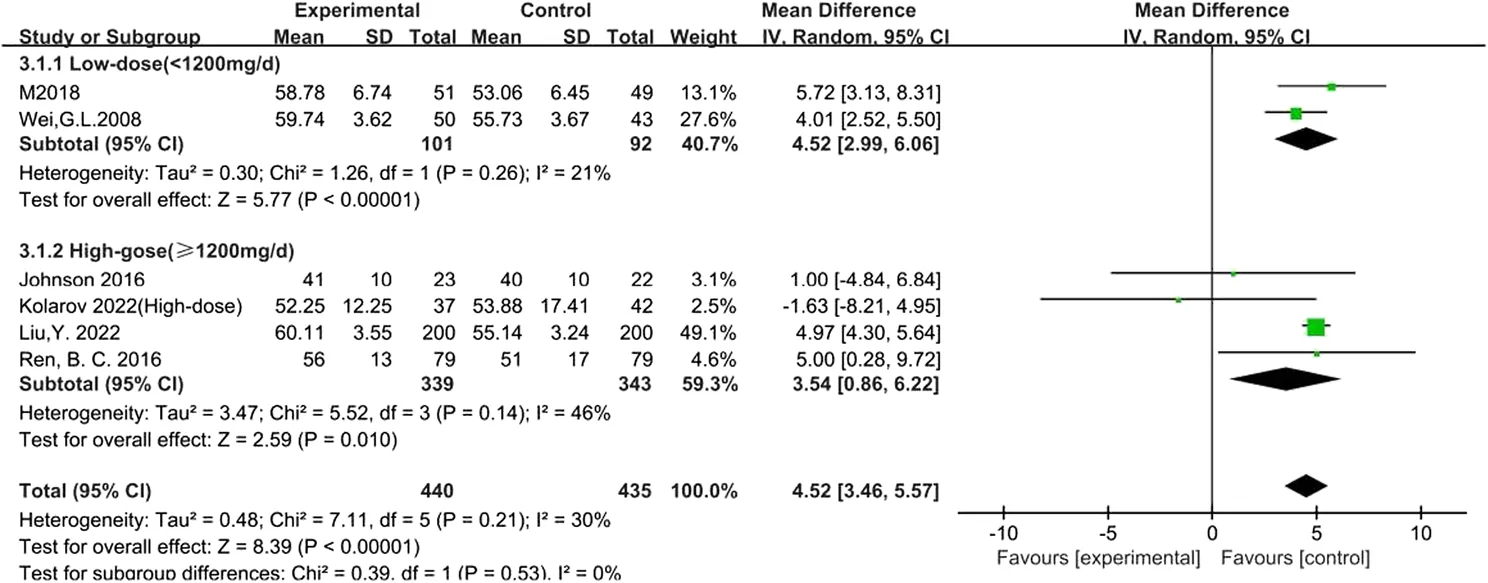
Sensitivity analysis of the effect of NAC on FEV_1_/FVC ratio in COPD patients (by dosage subgroup)

Subgroup differences were not statistically significant (Chi² = 0.89, *P* = 0.35), suggesting comparable efficacy across different doses in improving FEV_1_/FVC. (Fig. 7)

### Sensitivity analysis excluding AECOPD studies

To assess the robustness of our findings, we performed a sensitivity analysis excluding studies that enrolled populations not fully aligned with our target population of stable COPD. These included: the study by Liu et al. (2022) which enrolled COPD patients with concomitant lung cancer, and three studies that explicitly enrolled patients during acute exacerbations [16–18]. After excluding these four studies, the remaining studies were re-analyzed for each outcome.

**Fig. 8:**
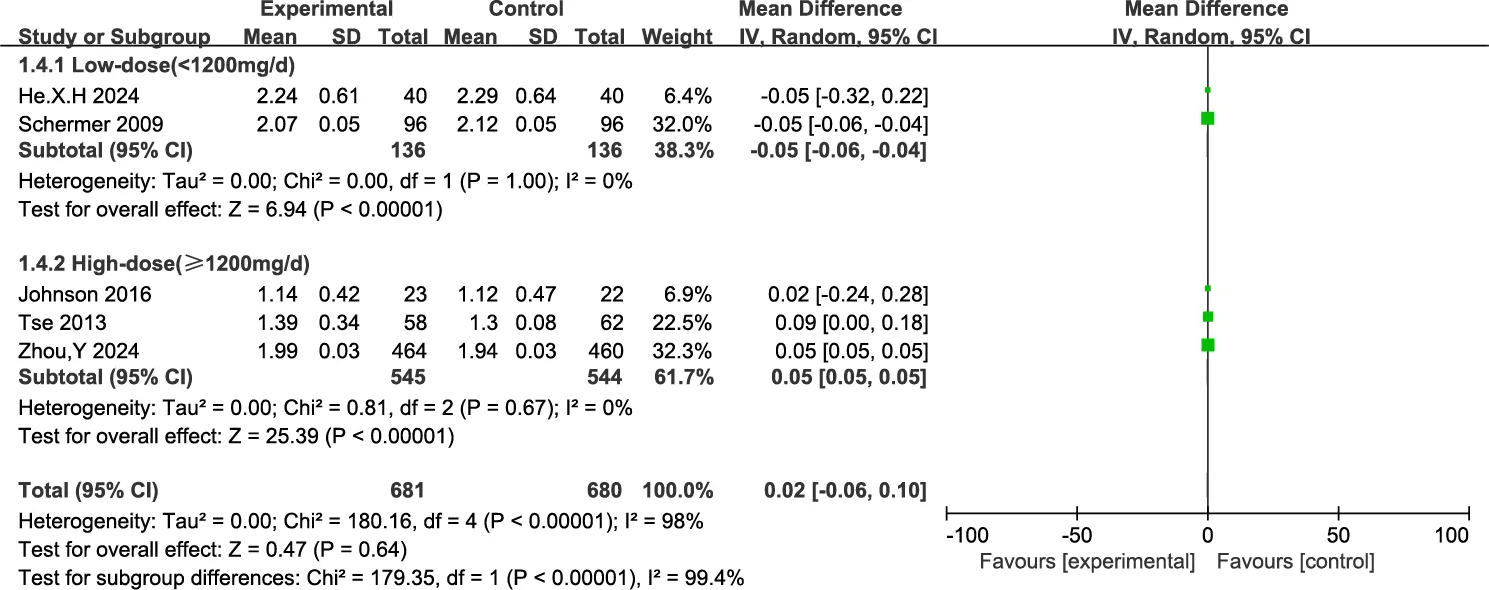
Sensitivity analysis of the effect of NAC on FEV₁ in COPD patients after excluding AECOPD studies (by dosage subgroup)

For FEV₁, after excluding AECOPD studies, five studies remained (Schermer 2009, He 2024, Johnson 2016, Tse 2013, Zhou 2024), with a total of 1,361 participants. The overall pooled effect was no longer statistically significant (MD = 0.05, 95% CI [-0.14, 0.24], *P* = 0.64), with substantial heterogeneity remaining (I² = 98%). Subgroup analysis showed that the high-dose group (≥ 1200 mg/day) maintained a statistically significant improvement in FEV₁ (MD = 0.05, 95% CI [0.05, 0.05], *P* < 0.00001, I² = 0%), while the low-dose group showed a significant negative effect (MD = -0.05, 95% CI [-0.06, -0.04], *P* < 0.00001, I² = 0%). The test for subgroup differences was statistically significant (*P* < 0.00001), confirming a dose-dependent effect.(Fig. 8).

**Fig. 9:**
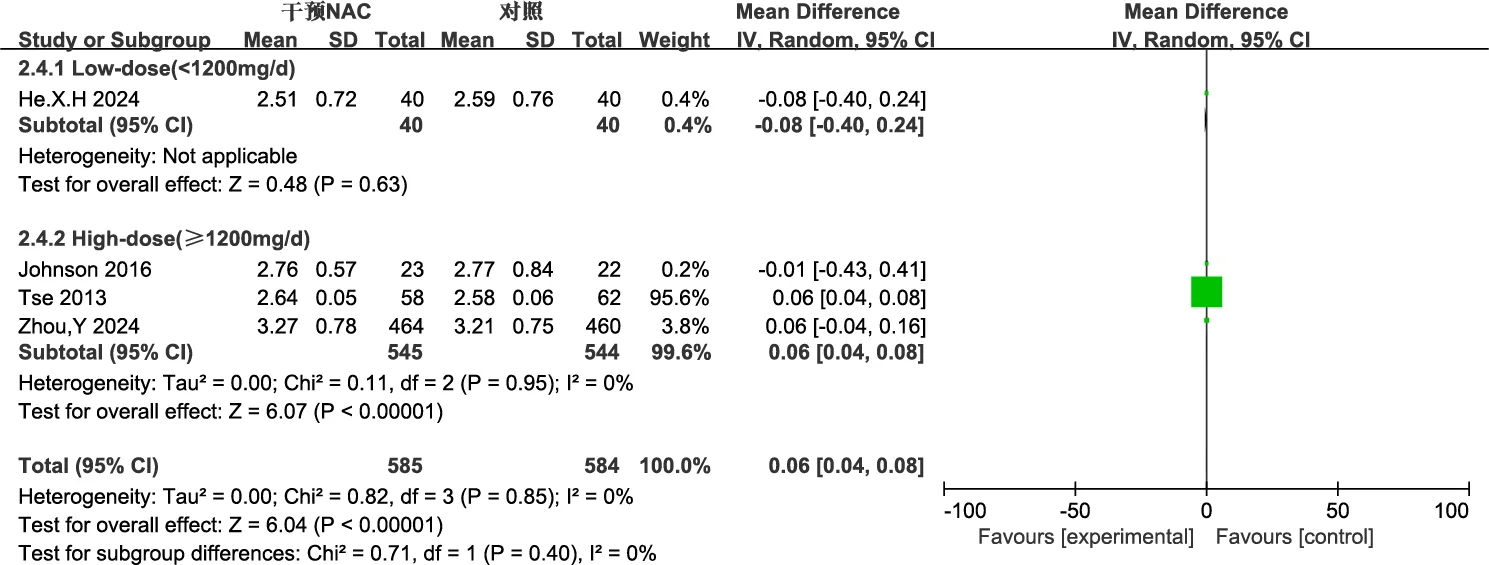
Sensitivity analysis of the effect of NAC on FVC in COPD patients after excluding AECOPD studies (by dosage subgroup)

For FVC, after excluding AECOPD studies, four studies remained (He 2024, Johnson 2016, Tse 2013, Zhou 2024). In the low-dose subgroup, only one study remained (He 2024), which showed no significant effect (MD = -0.08 L, 95% CI [-0.40, 0.24], *P* = 0.63); therefore, meta-analysis was not performed for this subgroup. The high-dose subgroup (three studies) showed a statistically significant improvement in FVC (MD = 0.06 L, 95% CI [0.04, 0.08], *P* < 0.00001, I² = 0%). The overall pooled effect across all four studies was also significant (MD = 0.06 L, 95% CI [0.04, 0.08], *P* < 0.00001), with no heterogeneity (I² = 0%).(Fig. 9).

**Fig. 10:**
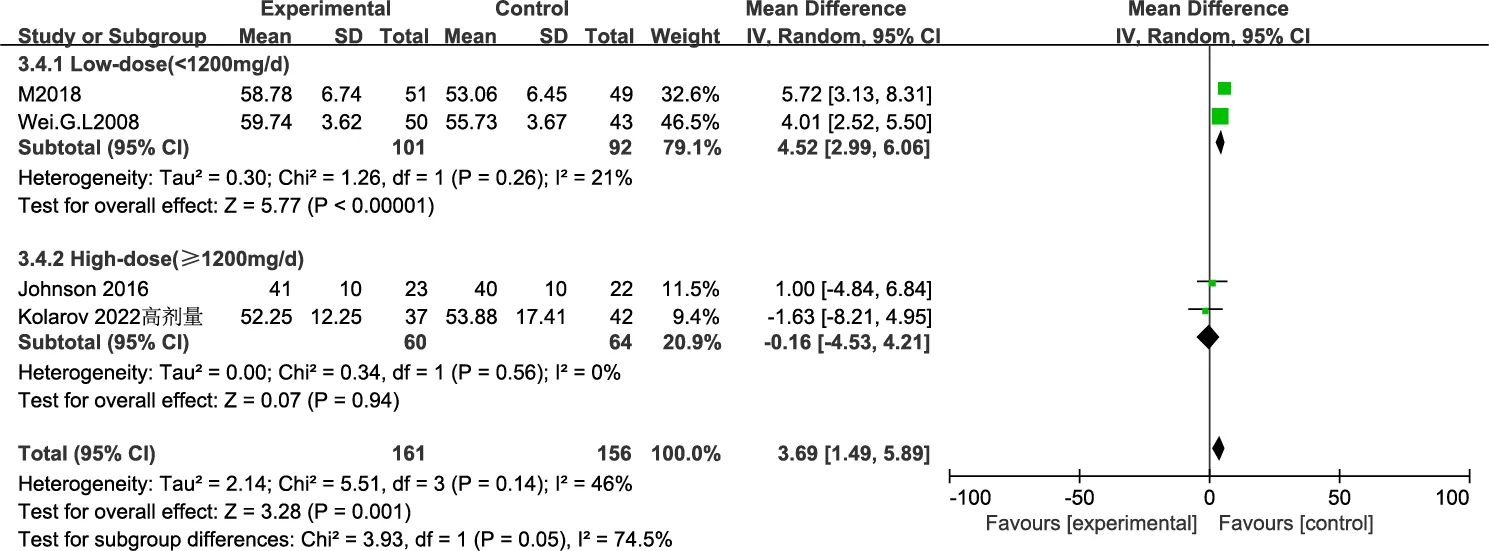
Sensitivity analysis of the effect of NAC on FEV₁/FVC ratio in COPD patients after excluding AECOPD studies (by dosage subgroup)

For FEV₁/FVC, after excluding AECOPD studies, four studies remained (Miao 2018, Wei 2008, Johnson 2016, Kolarov 2022). The low-dose subgroup (two studies) showed a significant improvement (MD = 4.52%, 95% CI [2.99, 6.06], *P* < 0.00001, I² = 21%). The high-dose subgroup (two studies) showed no significant effect (MD = -0.16%, 95% CI [-4.53, 4.21], *P* = 0.94, I² = 0%). The overall pooled effect was significant (MD = 4.52%, 95% CI [3.46, 5.57], *P* < 0.00001), with moderate heterogeneity (I² = 46%). The test for subgroup differences approached statistical significance (*P* = 0.05), suggesting a potential difference in treatment effects between doses.(Fig. 10).

These sensitivity analyses indicate that the beneficial effects of high-dose NAC on FEV₁ and FVC remained robust after excluding AECOPD studies, while the effect on FEV₁/FVC was driven primarily by low-dose NAC. The findings suggest that the effects of NAC on lung function may vary by dose in patients with stable COPD.

### Assessment of reporting biases

**Fig. 11:**
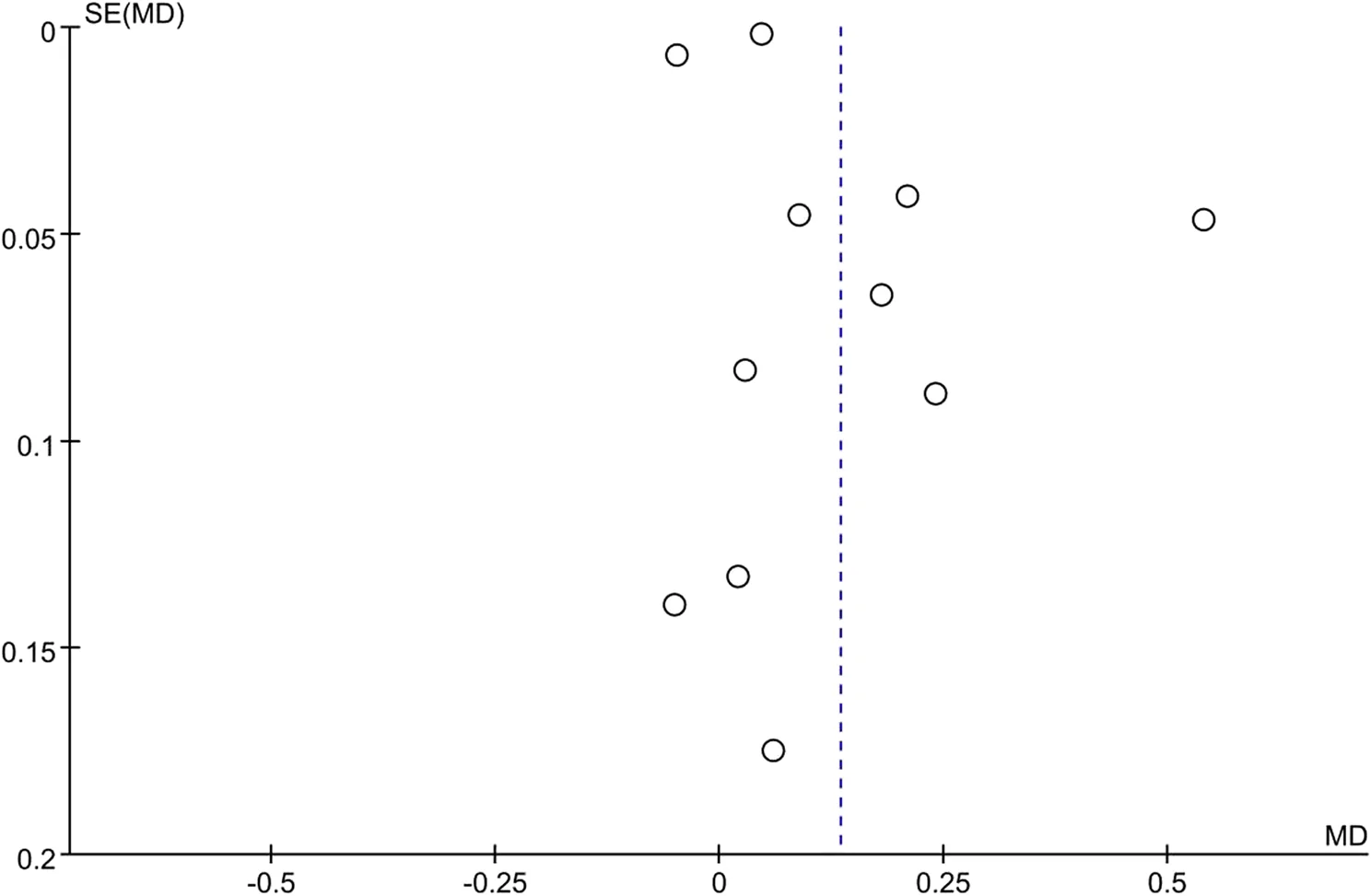
Funnel plot for FEV_1_

**Fig. 12:**
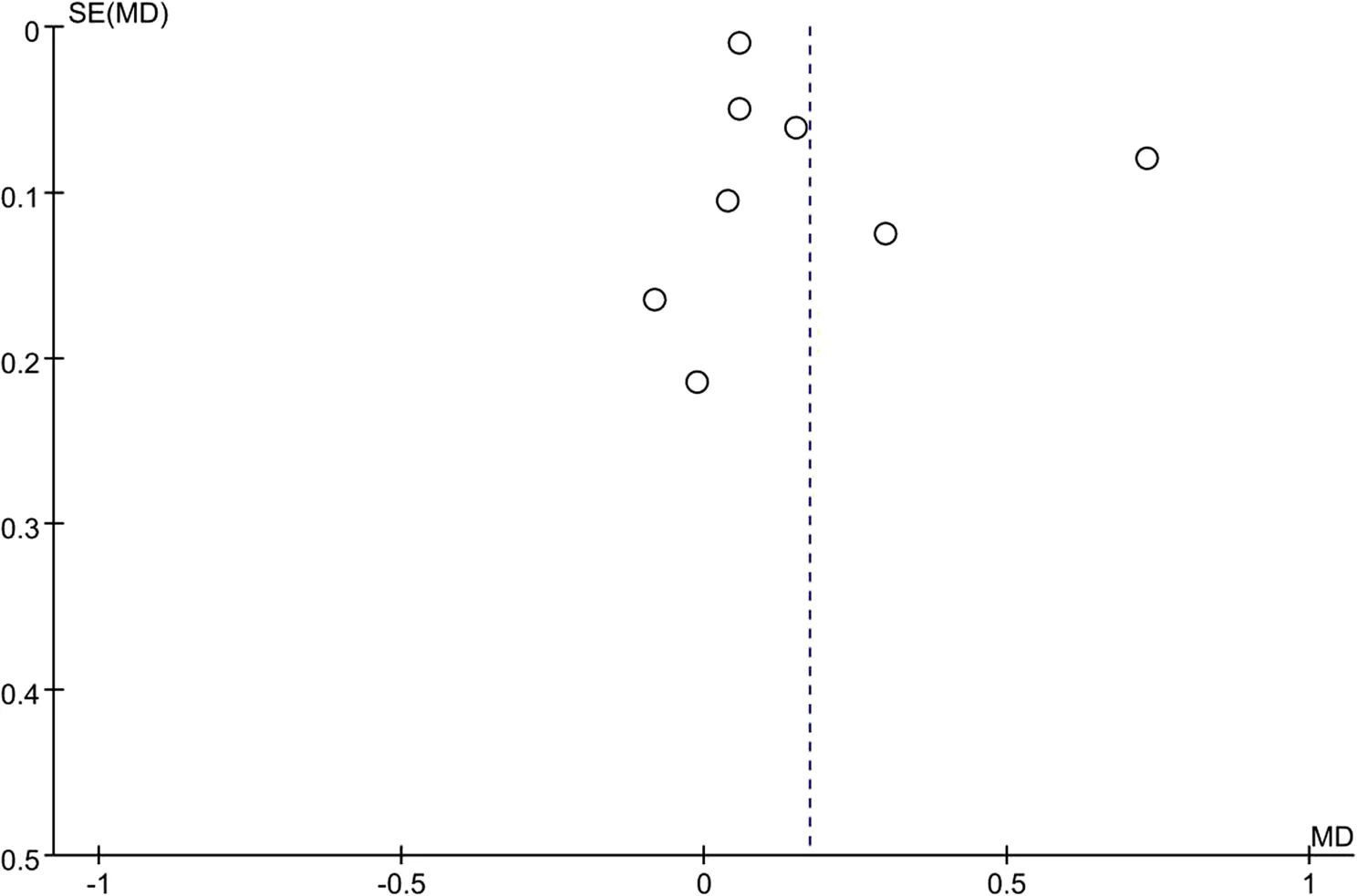
Funnel plot for FVC

**Fig. 13:**
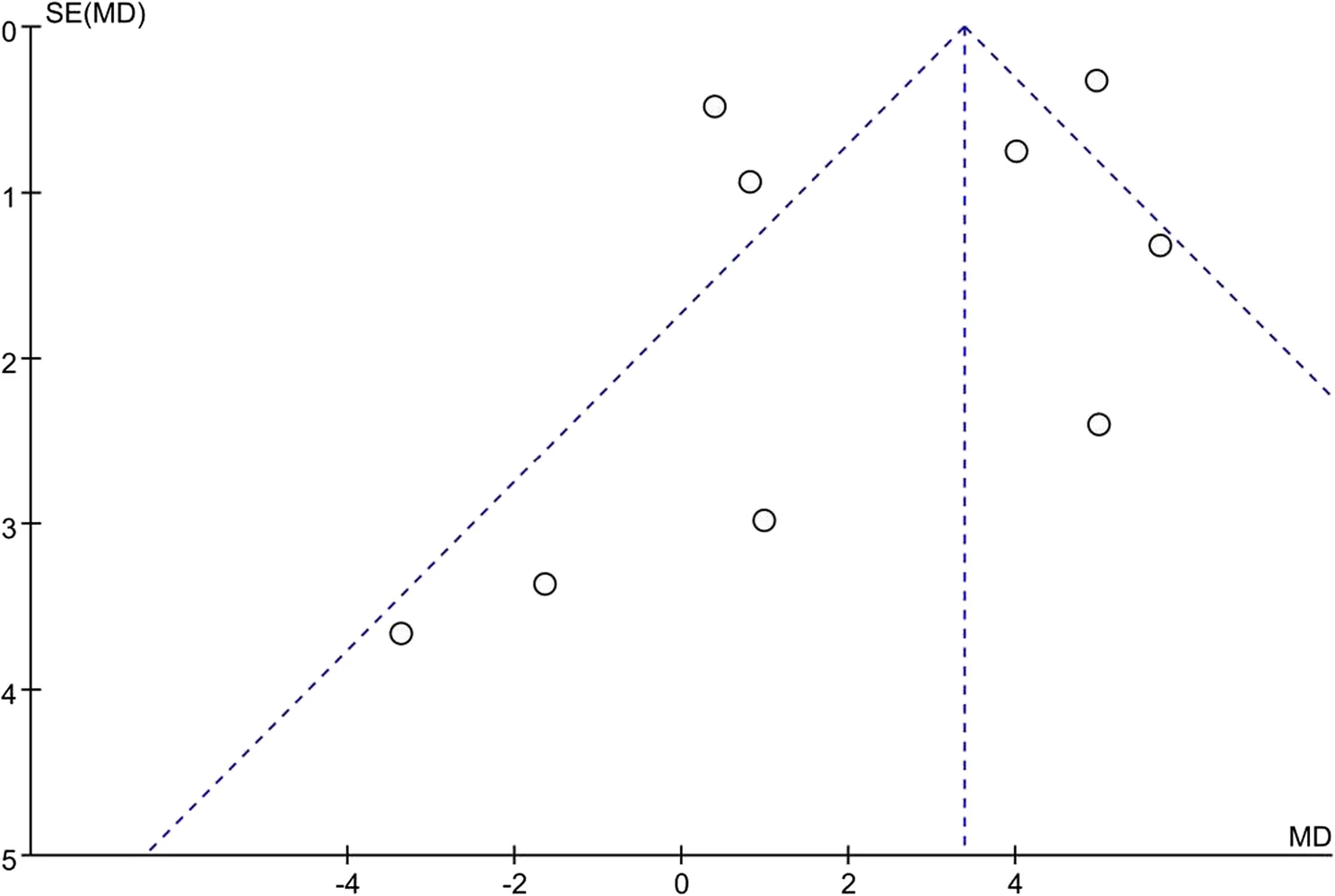
Funnel plot for FEV_1_/FVC

Visual inspection of the funnel plots for the primary outcomes (FEV₁, FVC) did not reveal obvious asymmetry(Figs. 11 and 12).However, the visual inspection of the funnel plot for the overall effect on FEV1/FVC showed potential asymmetry, with a sparse distribution of studies on the left side of the mean effect (Fig. 13). But, due to the limited number of included studies, the power to reliably detect publication bias was low, and formal statistical testing was not performed.

## Discussion

This meta-analysis systematically evaluated the effects of varying doses of N-acetylcysteine (NAC) on lung function in patients with chronic obstructive pulmonary disease (COPD). The findings indicate that NAC provides therapeutic benefits in improving pulmonary outcomes.

The dose-stratified analysis using 1200 mg/day as the cutoff was justified by prior evidence [10–14]. Our findings align with previous meta-analyses showing that high-dose (≥ 1200 mg/day), but not low-dose, NAC confers significant benefits in COPD outcomes [11–14].

Higher doses (> 1200 mg/day) were associated with more pronounced improvements in FEV₁ and FVC, whereas lower doses produced relatively greater enhancement in the FEV₁/FVC ratio [16, 19]. These results suggest that the effects of NAC on lung function may differ between high and low doses, highlighting the importance of individualized dosing strategies to optimize its antioxidant and mucolytic properties [3].

However, the meta-analysis revealed substantial heterogeneity in the results for FEV₁ (I² = 97%), FVC (I² = 91%), and FEV₁/FVC (I² = 90%), suggesting considerable variation across the included studies. These discrepancies may stem from differences in patient baseline severity, intervention protocols, treatment duration, and overall study quality [20]. For instance, some trials enrolled patients with GOLD stage II or higher, while others limited participation to those with mild to moderate COPD. Intervention methods also varied widely—including oral tablets, effervescent tablets, inhalers, and injections—with treatment durations ranging from 8 weeks to 12 months [21, 22]. Furthermore, most studies did not provide sufficient detail regarding blinding and randomization procedures, which may have introduced risks of selection and detection bias [23]. Variations in regional clinical guidelines and the use of concomitant therapies could also have influenced the outcomes [6, 24, 25]. Sensitivity analysis showed that excluding studies with high heterogeneity yielded more stable pooled estimates for FEV₁ and FVC, thereby strengthening confidence in the efficacy observed in the high-dose NAC group. Compared with previous studies, our research possesses the following innovative and systematic advantages:

On one hand, this study provides a dose-stratified analysis of NAC efficacy on pulmonary function in COPD patients. Previous meta-analyses have predominantly focused on NAC’s preventive effects against acute exacerbations or failed to refine dose-specific analyses. For instance, some studies evaluated only single doses (600 mg or 1200 mg/day) without exploring dose-response relationships with pulmonary function metrics.

On the other hand, our study incorporated a larger sample size (3176 participants) representing multinational populations, with a comprehensive analysis covering three core pulmonary function parameters: FEV_1_, FVC, and FEV_1_/FVC ratio. Subgroup analyses of high- and low-dose groups clearly demonstrated dose-response differences. On the contrary, most earlier meta-studies lacked structured stratified analysis, limiting the clinical guidance value of their conclusions.

Thus, our study offers a more systematic design, broader sample coverage, and deeper analytical perspectives, which collectively enhance the robustness of the evidence and allow for more precise theoretical guidance on individualized NAC dosing in COPD. Mechanistically, NAC replenishes glutathione (GSH) to strengthen antioxidant defenses in airway epithelium, suppresses NF-κB pathway activation, and lowers the expression of inflammatory mediators including IL-8 and TNF-α. It also reduces sputum viscosity, thereby facilitating mucus clearance [26]. Several reviews confirm that NAC’s antioxidant effects are dose-dependent [3, 27]. Higher doses are more likely to attain therapeutically effective concentrations in lung tissue, promoting GSH synthesis and enhancing radical scavenging capacity, which significantly alleviates oxidative stress and dampens inflammatory responses. Some reports further suggest that high-dose NAC results in greater cumulative exposure in the lungs, more effectively countering oxidative stress and mitigating airway remodeling and inflammation. This supports its superior clinical performance, especially in mucus-hypersecretion COPD phenotypes [28–30].

Beyond statistical significance, the clinical relevance of high-dose NAC on lung function should be considered. The minimal clinically important difference (MCID) for FEV₁ in COPD is generally accepted as 100 mL, as established by regulatory authorities and supported by multiple studies [1]. In our sensitivity analysis, high-dose NAC (≥ 1200 mg/day) improved FEV₁ by exactly 100 mL (MD = 0.10 L, 95% CI [0.03, 0.17]), reaching this clinically meaningful threshold. Although the lower bound of the confidence interval (30 mL) falls below the MCID, the point estimate suggests that the average patient may experience perceptible benefits. For FVC, the observed improvement (80 mL) was below the typical MCID of 200 mL cited in some studies [31], indicating a more modest effect. These findings suggest that high-dose NAC not only produces statistically significant improvements but also achieves clinically meaningful changes in FEV₁, supporting its role as a valuable adjunctive therapy in COPD management.

Nevertheless, our analysis has several limitations. First, certain RCTs failed to adequately describe randomization or blinding procedures, and variability in methodological quality may have introduced potential biase [23]. Second, differences in NAC formulations and bioavailability complicate direct comparisons of treatment effects [21, 22]. Third, inconsistencies in lung function measurement techniques and timing across studies could have led to measurement bias. Fourth, several trials were limited by small sample sizes and short follow-up periods, constraining insight into long-term outcomes. Finally, systematic reporting of adverse events and medication adherence was scarce, hindering a full assessment of safety and practicality in real-world settings [1].

These findings support that higher doses of NAC are associated with greater improvements in FEV₁ and FVC, suggesting a dose-related effect on lung function in COPD [32]. Owing to its dual mechanisms—antioxidant and mucolytic—high-dose NAC may represent a useful adjunct treatment for patients with stable COPD, particularly those with excessive sputum, high oxidative burden, or a history of exacerbations [33–35]. Clinically, dosing should be tailored based on disease severity, baseline lung function, and treatment adherence [36], moving away from a uniform approach toward personalized management.

To build upon these findings, several key questions demand attention in future studies.

Priority should be given to launching well-designed, multicenter RCTs that are sufficiently powered to definitively establish the optimal NAC dosing strategy tailored to distinct COPD phenotypes [28, 32, 37].

Equally important is a comprehensive assessment of safety profiles, including adverse events and patient adherence, which is crucial for managing the risks of long-term, high-dose NAC regimens [8, 17, 29].

Beyond traditional spirometry, future trials could strengthen efficacy evidence by incorporating objective biomarkers or imaging metrics, moving toward a more precision-based approach to therapy [25, 26, 34].

Finally, researchers should investigate whether specific patient subgroups—such as those with inherent antioxidant deficiency or a history of frequent exacerbations—derive particular benefit from NAC, thereby accelerating the development of personalized treatment protocols [5, 18, 30].

Sixth, the lack of stratified analysis based on follow-up duration may have influenced the reliability of our findings. As shown in Table 1, treatment duration varied considerably across included studies, ranging from 4 weeks to 36 months. Notably, there was an imbalance in follow-up duration between the low-dose and high-dose groups: studies in the low-dose group tended to have shorter follow-up periods (mostly 3–6 months), whereas high-dose group studies included both short-term (8–12 weeks) and long-term (≥ 12 months) trials. This disparity could potentially affect the observed effects, as longer follow-up may capture cumulative benefits of NAC therapy, particularly for outcomes like FEV₁ improvement which may require extended treatment to manifest clinically meaningful changes. Conversely, shorter follow-up periods might underestimate the true treatment effect. Although our sensitivity analysis partially addressed heterogeneity, we did not perform formal subgroup analysis by follow-up duration due to the limited number of studies in each category. Future meta-analyses with larger study pools should incorporate treatment duration as a stratification variable to better elucidate its impact on NAC efficacy.

Although studies enrolling AECOPD patients were included in the primary analysis, we acknowledge that this introduces clinical heterogeneity. A sensitivity analysis excluding these studies confirmed that the beneficial effects of high-dose NAC on FEV₁ and FVC remained robust, while the effect on FEV₁/FVC was primarily driven by low-dose NAC. Future studies should further investigate the differential effects of NAC in stable versus acute COPD populations.

## Conclusion

Our systematic review and meta-analysis evaluated the efficacy of varying doses of N-acetylcysteine (NAC) in patients with chronic obstructive pulmonary disease (COPD). The findings suggest that NAC confers therapeutic benefits in improving lung function, with high-dose regimens (≥ 1200 mg/day) demonstrating greater improvements in FEV₁ and FVC compared to lower doses. In contrast, lower doses appeared to exert more favorable effects on the FEV₁/FVC ratio. Considerable heterogeneity was observed across all pulmonary function outcomes, likely reflecting differences in patient characteristics, intervention protocols, and study design.

In clinical practice, high-dose NAC may be considered as adjunctive therapy for patients with stable COPD. Dosing should be individualized based on patient profiles and existing treatments, balancing potential benefits with tolerability and adherence.

Future research should prioritize well-designed, large-scale, multicenter randomized controlled trials using standardized protocols to further elucidate NAC’s mechanisms and dose-response characteristics across COPD subtypes. Long-term outcomes, alongside systematic evaluation of safety, adverse events, and medication adherence, will be essential to fully assess its real-world clinical utility.

## Supplementary Information

Supplementary Material 1.

## Data Availability

All data generated or analyzed during this study are included in this published article and its supplementary information files.
